# Sustained Spatial Attention to Vibrotactile Stimulation in the Flutter Range: Relevant Brain Regions and Their Interaction

**DOI:** 10.1371/journal.pone.0084196

**Published:** 2013-12-19

**Authors:** Dominique Goltz, Burkhard Pleger, Sabrina Thiel, Arno Villringer, Matthias M. Müller

**Affiliations:** 1 Department of Neurology, Max Planck Institute for Human Cognitive and Brain Sciences, Leipzig, Germany; 2 Institute of Psychology, University of Leipzig, Leipzig, Germany; 3 Clinic for Cognitive Neurology, University Hospital Leipzig, Leipzig, Germany; 4 Berlin School of Mind and Brain, Humboldt-University Berlin, Berlin, Germany; University Medical Center Goettingen, Germany

## Abstract

The present functional magnetic resonance imaging (fMRI) study was designed to get a better understanding of the brain regions involved in sustained spatial attention to tactile events and to ascertain to what extent their activation was correlated. We presented continuous 20 Hz vibrotactile stimuli (range of flutter) concurrently to the left and right index fingers of healthy human volunteers. An arrow cue instructed subjects in a trial-by-trial fashion to attend to the left or right index finger and to detect rare target events that were embedded in the vibrotactile stimulation streams. We found blood oxygen level-dependent (BOLD) attentional modulation in primary somatosensory cortex (SI), mainly covering Brodmann area 1, 2, and 3b, as well as in secondary somatosensory cortex (SII), contralateral to the to-be-attended hand. Furthermore, attention to the right (dominant) hand resulted in additional BOLD modulation in left posterior insula. All of the effects were caused by an increased activation when attention was paid to the contralateral hand, except for the effects in left SI and insula. In left SI, the effect was related to a mixture of both a slight increase in activation when attention was paid to the contralateral hand as well as a slight decrease in activation when attention was paid to the ipsilateral hand (i.e., the tactile distraction condition). In contrast, the effect in left posterior insula was exclusively driven by a relative decrease in activation in the tactile distraction condition, which points to an active inhibition when tactile information is irrelevant. Finally, correlation analyses indicate a linear relationship between attention effects in intrahemispheric somatosensory cortices, since attentional modulation in SI and SII were interrelated within one hemisphere but not across hemispheres. All in all, our results provide a basis for future research on sustained attention to continuous vibrotactile stimulation in the range of flutter.

## Introduction

If you try to find your muted but vibrating cellphone in the depth of your handbag, attention is paid continuously to the various tactile sensations of your hand. The distance between other objects in your handbag and the cellphone determines how strongly the objects transmit the vibration, and sustained attention to this information is crucial to finally locate the cellphone. In previous literature, tactile attention has often been investigated in blocks in which (few) transient stimuli with relatively long inter-stimulus intervals were presented that allowed the underlying generators to recover [1-3]. Transient stimuli with long inter-stimulus intervals are useful to study post stimulus processing; however, there are some shortcomings with this approach when studying attention. For example, if multiple transient stimuli are simultaneously presented to one hand, it is difficult to disentangle neural activity from the to-be-attended and the to-be-unattended stimulus. This is especially a problem for electroencephalography (EEG) studies. Additionally, the aforementioned example clearly shows that in everyday life, deployment of attention to a certain stimulus is not only restricted to stimuli that are just present for a few milliseconds. Rather, attention often has to be focused for several seconds on stimuli that are continuously present.

In recent EEG studies, we tried to circumvent these limitations of transient stimulation by using an alternative approach that is applicable to diverse sensory modalities. We mimicked the condition of sustained attention to long-lasting sensory input by applying continuous stimuli at a certain frequency [4-7]. In EEG, such frequency-tagged stimuli elicit the steady state evoked potential (SSEP), an oscillatory response of the same frequency as the applied driving stimulus that can easily be analyzed in the frequency domain [8]. Crucially, one can analyze attentional deployment to frequency-tagged stimuli because paying attention to such a stimulus causes an increase in SSEP amplitude compared to when the respective stimulus is unattended [9,10]. One of the advantages over transient stimuli is that if several stimuli of the same sensory modality are concurrently presented, it is still possible to disentangle their neural responses as long as each stimulus is tagged with an individual driving frequency. In addition, given that one measures a continuous oscillatory signal, temporal neural dynamics of attentional shifts can be analyzed as well [11,12]. In previous studies on visual, auditory, and intermodal attention, we were able to investigate early perceptual processes and the neural dynamics of attention in the human brain [6,13,14]. Moreover, mechanisms that generate SSEPs and the underlying cortical generators could be uncovered [14,15]. These findings clearly show that frequency-tagged stimuli provide an innovative way to study sustained attention on continuous sensory stimuli.

In somatosensation, continuous stimuli play a major role in diverse fields. For example, continuous vibrotactile stimulation has been used in animal studies to explore the neural responses in the sense of flutter; that is to say, between 20 and 50 Hz [16-18]. These studies have provided fundamental physiological knowledge ranging from skin mechanoreceptors that code flutter and other vibrotactile stimuli [19,20] to the responses of single cells and local field potentials to these stimuli in primary somatosensory cortex (SI) and beyond [16,21-24]. Furthermore, continuous vibrotactile stimulation has often been used in human functional magnetic resonance imaging (fMRI), transcranial magnetic stimulation (TMS), and EEG studies to examine tactile working memory [25-28], tactile decisions [29] or detection tasks [30].

In previous EEG studies, we applied vibrotactile stimulation at a frequency range of 20 Hz concurrently to left and right index fingers and asked participants to pay attention to one finger, while ignoring the other one [31-33]. Similar to what we found in the visual and auditory modality [5,6,13], the amplitude of the somatosensory SSEP (SSSEP) was significantly increased when subjects attended to one location that was stimulated at a certain frequency compared to when that body location was unattended. Source analysis revealed that the attentional modulation of the SSSEP originates from contralateral SI [32]. Yet, spatial resolution and volume conduction limits the validity of estimated cortical sources. Besides, we needed to stimulate different body locations with different frequencies in order to be able to distinguish the neural responses in the analysis. An inherent confound related to that necessity is, that subjects might have adopted a strategy to attend to stimulation frequency rather than to location (but see Müller et al. [34] to exclude that possibility in a corresponding visual experiment). This in turn may have promoted the focused SI modulation pattern.

Contrary to the aforementioned approach and as already mentioned above, other studies investigating tactile attention often presented (blocks of few) transient stimuli with a relatively long inter-stimulus interval [1-3,35,36]. Given that the inter-stimulus intervals often exceeded one or even several seconds [1-3], there was often no control over whether subjects constantly focused on the to-be-attended stimulus. Accordingly, sustained attention to longer lasting (> 1 s) or permanent stimuli was left rather unexplored. Imaging studies investigating sustained attention with continuous (vibro-) tactile stimuli are generally more frequent than respective EEG or magnetoencephalography (MEG) studies ([37,38], but see for example Eimer et al. [39]); still, they are relatively rare and resulted in a rather inconclusive picture. Reviewing all these studies on tactile attention, several striking discrepancies can be noted: First, there seems to be a tremendous difference in whether and to what degree activity in SI is modulated by attention. One of the earliest studies on tactile attention was done with intracranial recordings in monkeys: Hyvärinen and colleagues (1980) revealed that only 16% of the recorded neurons in SI increased their activity when attention was directed to the vibrotactile input [40]. In contrast, a later single cell recording by Hsiao and colleagues (1993) showed that about 50% of the SI neurons increased their activity with attention [41]. Focusing on human EEG or MEG studies, some studies report attentional modulation of early components of the somatosensory evoked potential (SEP) that clearly originate from SI [3]. In contrast, other studies reported attentional modulation only in a later time window (> 100 ms post-stimulus) corresponding to the activity in secondary somatosensory cortex, SII [1,3,42,43]. Yet, it should be noted that these studies only claim that this is the last point in time when attentional modulation becomes significant. Nonetheless, imaging studies also showed diverse findings. In several studies, modulation of SI was either not present [2,44], failed to reach significance, or was simply smaller than in SII [38,45,46]. Still, other studies found a significant increase in activity in contralateral SI that was of equal magnitude to that of SII [35,36]. Nonetheless, this robust attentional modulation effect in SI seems to be rather rare and that is why Johansen-Berg and Lloyd (2000) hypothesized that it strongly depends on an *active* distractor task [47]. They assumed that if an attend touch condition is compared to a passive ignore condition, a significant attentional modulation in SI is less likely. However, if the attend touch condition is compared to an active distractor task, like a mental arithmetic task or a visual distraction task, this active task will better control and direct participants’ attention away from the tactile input, resulting in a stronger modulation in SI. Interestingly, only a few researchers have thought of using an active distractor task within the tactile domain [3,48,49]. However, attention can be easily switched between hands in a spatial attention approach, so that attention to the left hand can serve as an active tactile distraction task for the respective right hand condition (and vice versa). In these studies, attentional modulation was basically revealed in SI, but additionally depended on further experimental factors such as stimulation intensity as well as the temporal characteristics of attentional deployment [3,48]. Besides, participants were presented with (blocks of) transient stimuli with a relatively long inter-stimulus interval (~1s) instead of continuous stimulation. Yet, in everyday life tactile experience is better mimicked by *sustained* tactile input to both hands. Given this inconsistency of electrophysiological and brain imaging data and the rare application of spatial attention within the tactile modality, it is of particular interest whether *sustained spatial attention* to *continuous* tactile stimulation can modulate somatosensory processing as early as in SI. Moreover, it is of interest *which* areas of SI contribute to the attentional modulation of the measured signals.

SI comprises four cytoarchitectonically different areas, BA 1, 2, 3a, and 3b (for a detailed description see 50-52). To the best of our knowledge, only the study by Hyvärinen and colleagues (1980) tried to localize the attention effects within SI ([40], see also the study of Burton et al. [38] although they found no significant SI modulation). From the 16% of SI neurons that increased their activity with attention, most of the neurons were located in BA 1, whereas the minority was located in BA 3a/3b. Predominantly by studies in nonhuman primates, it has been shown that the four cytoarchitectonically different SI areas also show functional diversity [53]. Area 3b in the rostral wall of the postcentral sulcus receives mainly cutaneous input from both rapidly and slowly adapting receptors, and its representation of the body parts is highly interconnected to the representation in area 1 [50,51]. Area 1 on the vertex of the postcentral gyrus receives predominantly afferents from rapidly adapting cutaneous receptors [50,51]. The receptive fields of neurons in area 1 are much larger and have a more complex response property than the neurons in area 3b [50]. Caudal to area 1 lies area 2, which receives input from cutaneous receptors as well as proprioceptors in muscles and joints [50,53]. Crucially, in contrast to area 3b and 1, area 2 is sensitive to more complex cutaneous stimuli or active tactile discrimination tasks [54-56]. Deep within the central sulcus lies BA 3a. It receives primarily proprioceptive information from receptors in muscle spindles and joints [50]. With respect to the way the information is relayed to cortex, most thalamic information is projected to BA 3b and 3a. BA 1 and 2 receive much less direct thalamic information but receive their main information from BA 3a and 3b [50,53]. Additionally, there are further connections between the four different Brodmann areas, and also to other cortical areas such as the primary motor cortex, giving each of these areas its individual connection profile [50,53]. Nevertheless, one common connection is that all of the four Brodmann areas project to SII [50,53]. Given the fact that the SI subdivisions are connected differently and linked to the processing of different somatosensory information (either cutaneous and/or proprioceptive), it is important to have a closer look at the area-specific SI activation.

Another inconsistency in previous fMRI studies on sustained tactile attention becomes visible with respect to the *activation pattern* in brain areas showing tactile attentional modulation. Generally, most studies have revealed that attention significantly increases activity in SI contralateral to the to-be-attended hand as well as in SII and insula in both cortical hemispheres [36,45,46]. However, especially with respect to SII and insula, a more recent study by Sterr et al. (2007) challenged this view by showing that attention has no effect on insula and that it influences SII only ipsilaterally to the attended hand [35]. Interestingly, the effect on ipsilateral SII was driven by a mixture of both an increased activity in the attend condition and a decreased activity below baseline in the respective control condition (see also Burton et al. [38]). In contrast, contralateral SII activity was present in all participants but no difference in signal intensity was revealed between both conditions. As there are few studies so far using a tactile distraction task, it is of particular interest to explore the activation pattern in attention modulated brain areas in more detail in such experimental paradigms.

Finally, in all of these studies the relationship between the attention effects in SI, SII, and insula remains rather untouched. Yet, it is well known that SI and SII are directly interconnected [50] and that SII in turn projects to insular cortex [57]. Given these anatomical connections it seems very likely that attentional modulation in one region has an influence on neural activity in connected regions as well.

In the present fMRI study, we adopted the experimental paradigm from our previous EEG studies [31,32] by manipulating attention between hands to further investigate cortical activation patterns during *sustained spatial attention*. In particular, we focused on three research questions: First, can we validate attentional modulation in SI and, if yes, in which subregion of SI is the attention effect especially pronounced? Second, what is the activation pattern in SI, SII, and insular cortex? Finally, is there a direct linear relationship between the attention effects in SI, SII, and insular cortex? We applied vibrotactile stimulation at a rate of 20 Hz for 3 s to both index fingers simultaneously. Participants were cued in a trial-by-trial fashion to attend to either the left (AL) or right hand stimulation (AR), and to detect rare target events embedded in the stream. We revealed that attention modulated BOLD activity in SI and SII, contralateral to the to-be-attended-hand. In SI, attentional modulation was most prominent in BA 1, 2 and 3b. Moreover, attention to the right (dominant) hand resulted in additional modulation in left posterior insula. Interestingly, all of the effects were driven by an increased activity in the respective attend condition (i.e., paying attention to the contralateral hand), except for the effects in left SI and insular cortex. In left SI, the effect was related to a slight increase in activation when attention was paid to the contralateral hand as well as a slight decrease in activation when attention was paid to the ipsilateral hand (i.e., the tactile distraction condition). In contrast, the effect in left posterior insula was exclusively driven by a relative decrease in activation in the tactile distraction condition (AL). Finally, we found that attention effects within one hemisphere but not across hemispheres were correlated with each other.

## Methods

### Ethics Statement

The study was approved by the Ethics Committee of the University of Leipzig, and participants gave written informed consent prior to the experiment.

### Participants

Twenty-three volunteers between the age of 21 and 30 years (mean age = 25 years, standard deviation (SD) = 2.6) were recruited as participants. Handedness was assessed by the Edinburgh Handedness Inventory [58] and only right-handed participants with a laterality quotient (LQ) ≥ 70 were included (mean LQ = 95.2, SD = 8.5). All participants reported normal or corrected-to-normal vision, and no participant had a history of neurological or psychiatric disorder. The data of three participants were excluded from further analyses due to technical problems with the response pad. One additional participant was excluded due to poor performance (detection rate below 25% in both conditions). Thus, 19 participants (8 female; mean age = 25 years, SD = 2.8; LQ = 95.7, SD = 7) were included in the analyses.

### Stimuli and Apparatus

**Figure 1:**
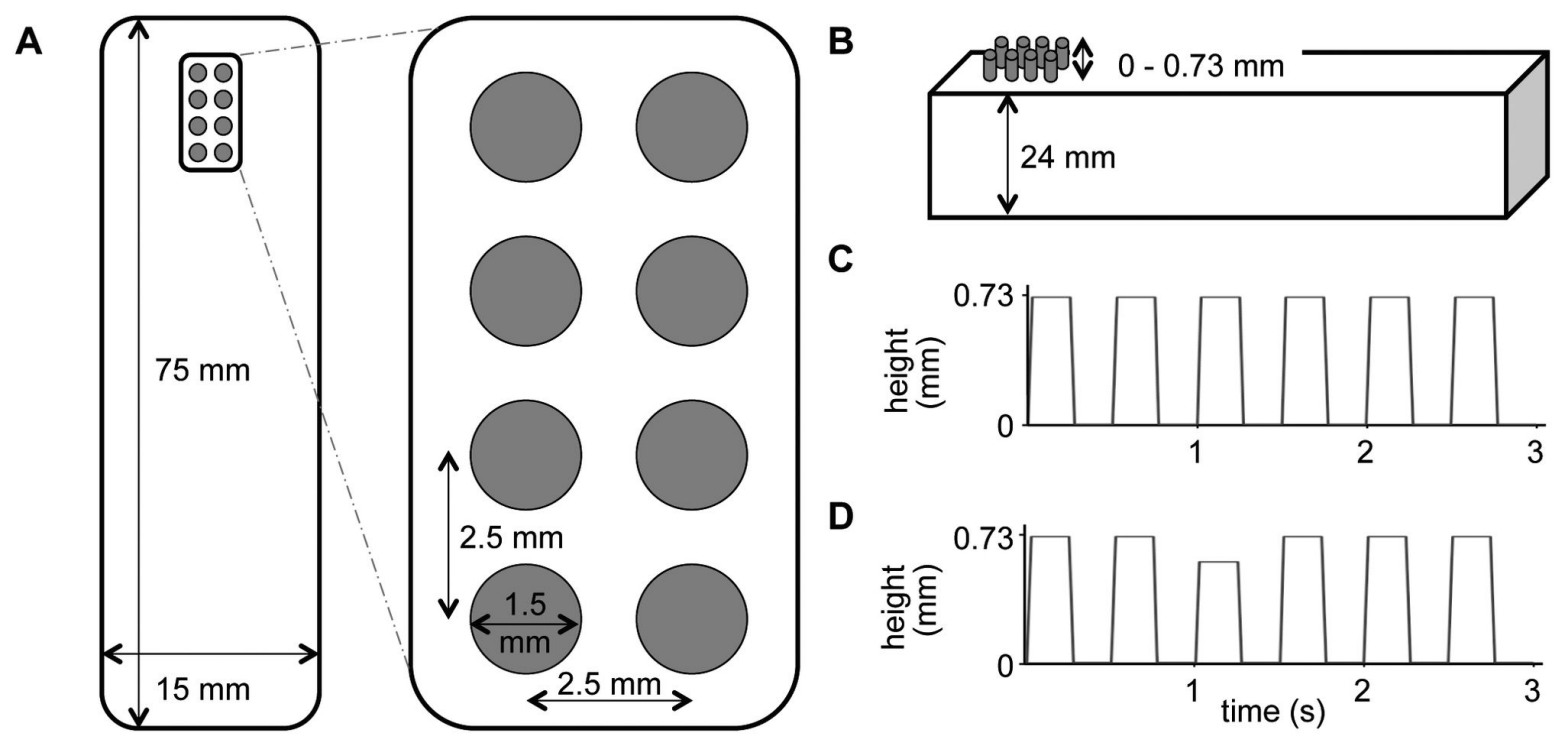
Schematic of stimulation device and target-absent and target-present trials. Figure **A** depicts a top view of the stimulation device for one finger. The precise composition of one 8-pin stimulation surface is visible in the close-up. Note that the left and right index fingers were stimulated by such a stimulation device. Figure **B** shows a lateral sketch of the stimulation device. During the tactile stimulation, all pins change their height between 0 and 0.73 mm. Figure **C** represents a simplified 3 s square-waved vibrotactile stimulation, which was predominantly presented to the left and right index finger. In contrast, Figure **D** represents a simplified target-present stimulation. Note that all 8 pins of the stimulation device presented either the target-absent stimulation (C) or the target-present stimulation that felt like little temporal gaps within the stimulation (D). For display purposes, the 20 Hz stimulation is shown as 2 Hz.

Participants were stimulated with a vibrotactile stimulation stream of 20 Hz simultaneously on their left and right index finger. The stimulation lasted for 3 s, and for each finger the stimulation was applied through an 8-dot piezo-electric stimulation display (2 × 4 quadratic matrix, 2.5 mm spacing; Piezostimulator; QuaeroSys, Schotten, Germany; see Figure 1A and B for a schematic view of the stimulation device). All pins moved simultaneously in a square-waved fashion between 0 (all pins down) and 0.73 mm (all pins up) (see Figure 1B). The duration of the up- and down-state was the same, lasting 25 ms. Participants were instructed to detect rare targets at the to-be-attended side that felt like little temporal gaps within the stimulation. These targets were variable attenuations in stimulation amplitude (i.e., maximal pin extension was randomly lowered by 15% or 20%) and appeared at different points in time during the stimulation (either after 1 s or 2 s). They lasted for 150 ms and appeared maximally once within a trial. A trial incorporating a target was called target-present trial (2/7 of all trials); all other trials were called target-absent trials. Note that targets were only presented on the to-be-attended to side. Schematic trials for target-absent and target-present stimulation are shown in Figure 1C, and D, respectively. The stimulation sequence was programmed by Presentation® software (version 14.9, Neurobehavioral Systems, Inc.).

### Procedure and Design

A trial started with a 2 s presentation of a white arrow on a black screen that indicated the to-be-attended hand for the upcoming trial. Subsequently, the vibrotactile stimulation was presented for 3 s. During this time participants were asked to fixate a white cross in the center of the black screen. Participants had to pay attention to the cued side and press a button with their right foot as soon as they detected a target. Due to the complex experimental setting, we instructed participants to focus on accuracy and not on speeded response. The inter-trial interval varied between 1 and 4 s. During this time a central fixation cross was presented. Each participant was familiarized with the task prior to the experiment. The experiment consisted of one session with 140 trials. Trials were equally divided into attend-left and attend-right hand conditions. For each condition, 20 target trials were presented randomly. The order of trials was pseudo-randomized with the constraint of no more than three successive trials in the same condition.

### fMRI Data Acquisition

Imaging was performed using a 3 T Magnetom Trio MRI System (Siemens, Erlangen, Germany) equipped with a standard 12-channel head coil. A cushion was used in order to minimize participants’ head motion, and a field map measured the distortions of the magnetic field. BOLD (blood oxygen level dependent) sensitive images were collected using a T2*-weighted echo-planar imaging (EPI) sequence (repetition time (TR) = 2000 ms, echo time (TE) = 30 ms, flip angle = 90°). The acquired matrix was 64 x 64 with a field of view (FOV) of 19.2 cm, resulting in an in-plane resolution of 3 mm x 3 mm. The slice thickness was 4 mm with an interslice gap of 1 mm. Thirty slices were acquired in an interleaved mode. The scanning planes were oriented according to the anterior commissure - posterior commissure (AC-PC) convention. For each participant, one run of 544 volumes was obtained. Anatomical images were previously recorded in a separate session using a T1-weighted 3D magnetization prepared rapid gradient echo (MP-RAGE) sequence (TR = 1300 ms; TE = 3.93 ms; flip angle = 10°; inversion time (TI) = 650 ms; image matrix = 256 x 240; spatial resolution = 1 mm x 1 mm x 1.5 mm). 

### Data Analyses

#### Behavioral data

Behavioral responses were considered as correct if a button press occurred in a target-present trial. False alarms were defined as a button press in target-absent trials. Accordingly, the percentage of correct responses (detection rate) and of false alarms was calculated. For each of these quantities, the values for the conditions AL and AR were compared by means of paired two-sided t-tests. Reaction time data were not analyzed due to a focus on accuracy.

#### fMRI Data

MRI data were analyzed using SPM 8 (Wellcome Trust Centre for Neuroimaging, UCL, London, UK). The initial three functional volumes were discarded in order to allow longitudinal magnetization to reach equilibrium. The remaining functional volumes were slice time-corrected using the middle slice as reference, realigned to the first image of the time series, and corrected for movement-induced image distortions (6-parameter rigid body affine realignment). In order to account for magnetic field inhomogeneities, a distortion correction based on the field map measurement was applied. Thereafter, functional and anatomical images were co-registered. Anatomical images were then segmented into gray and white matter as well as cerebrospinal fluid (CSF) and normalized to a standard stereotaxic space using the T1 template by the Montreal Neurological Institute (MNI) delivered with SPM. The normalization parameters were then applied to the functional EPI series. Spatial smoothing was performed on the functional data using a three-dimensional Gaussian filter of 8 mm full-width at half-maximum (FWHM).

Voxelwise statistical analyses were calculated using the General Linear Model. Four regressors were specified: (1) target-absent trials in which participants were asked to pay attention to the left hand stimulation (AL), (2) target-absent trials in which participants were asked to pay attention to the right hand stimulation (AR), (3) target-present trials for AL, and (4) target-present trials for AR. Since we were not interested in the processing of targets, we only focused on the effects of target-absent AL and target-absent AR, and we compared these two active tactile attention conditions with each other. Predictors of the hemodynamic response were modeled by a stick function, placed at the onset of the vibrotactile stimulation. The stick function was convolved with a canonical hemodynamic response function (event-related analysis). A temporal high pass filter of 128 s was used in order to remove low-frequency drifts. We performed random-effects analyses by first contrasting target-absent AL with target-absent AR and vice versa on single subject level. These individual contrast images were then entered into a second-level analysis using one-sample t-tests. The resulting t-maps were thresholded at p < 0.001 uncorrected, with the extent threshold of k >= 10 as cluster size. Small volume corrections were applied to independent a priori defined regions of interest (ROI), left and right SI, SII, and insula for AR and AL, respectively. To this end, we created anatomical ROI masks from probabilistic cytoarchitectonic maps using the SPM Anatomy Toolbox (version 1.7; [59]). Cluster of BOLD activity surpassing a threshold of p <0.05 (FWE-corrected; cluster-level) were considered as significant. All reported coordinates correspond to the anatomical MNI space as used in SPM 8. The probability maps of the Anatomy Toolbox were used for assignment of BOLD responses to their underlying Brodmann Areas (BAs). Maximal activation refers to voxel with the highest statistical t-value in the respective brain region.

Correlation analyses were performed to examine a possible relationship between activity in left and right SI, SII, and insula. For this, we took those activation clusters (p < .001) that overlapped with our a priori defined masks and extracted the first eigenvariate for each of these masks for each participant. Note that the eigenvariate values are derived from singular value decomposition and effectively provide weighted mean activity where atypical voxels are downweighted. Thereby, eigenvariate values are more robust to heterogeneous activity within a cluster than mean values (see 27,60,61 for further analyses using eigenvariate values). As a result, we obtained a weighted mean activity value for each participant for each brain area showing an attention effect. The eigenvariate values of the different brain areas were then subjected to correlation analyses using Pearson’s r or Spearman’s rho for normally or non-normally distributed data respectively. Consequently, correlation analyses were performed for the following brain areas: left SI and left SII, left SII and left insula, right SI and right SII, left and right SI, as well as left and right SII. Following that, the correlation coefficient r was squared (R^2^) and converted to percentage. This allowed us to evaluate the amount of shared variability.

## Results

### Behavioral Data

Across all participants and conditions, 61.32% of targets were detected with a mean false alarm rate of 0.04%. The mean detection rate for AL was 63.68% (standard error of the mean (SE) = 4.84%) with a false alarm rate of 0.03% (SE = 0.01%). For AR, 58.95% (SE = 5.31%) of target events were detected and participants pressed incorrectly in 0.04% (SE = 0.02%) of target-absent trials. There was neither a significant difference between the two conditions in detection rate (*t*(18) = .71, p = .487) nor in false alarm rate (*t*(18) = -.74, p = .466).

### fMRI Data

A summary of all activated brain areas can be seen in Table 1. Results reported in the following paragraphs refer to our a priori defined ROIs.

**Table 1:** Anatomical locations and statistical information on all activated clusters after small volume correction.

						**MNI coordinates**		
**Contrast**	**Anatomical region**	**Area**	**Cluster size**	**Cluster-level *p*_FWE-corr_**	**Peak-level t value**	**x**	**y**	**z**
AL vs. AR	Right SI	1	386	< 0.001	7.38	54	-19	49
	Right SII	OP 1	115	< 0.001	8.00	48	-16	13
AR vs. AL	Left SI	2	297	< 0.001	6.99	-45	-28	49
	Left SII	OP 1	122	< 0.001	5.41	-48	-22	16
	Left Ins	Lg 2	23	0.007	5.73	-33	-19	10

Abbreviations: SI = Primary Somatosensory Cortex, SII = Secondary Somatosensory Cortex, Ins = Insula, Lg= Granular Layer of Insula, OP = Parietal Operculum, MNI = Montreal Neurological Institute, FWE-corr = family-wise error corrected

#### Effects of paying attention to the left index finger

**Figure 2:**
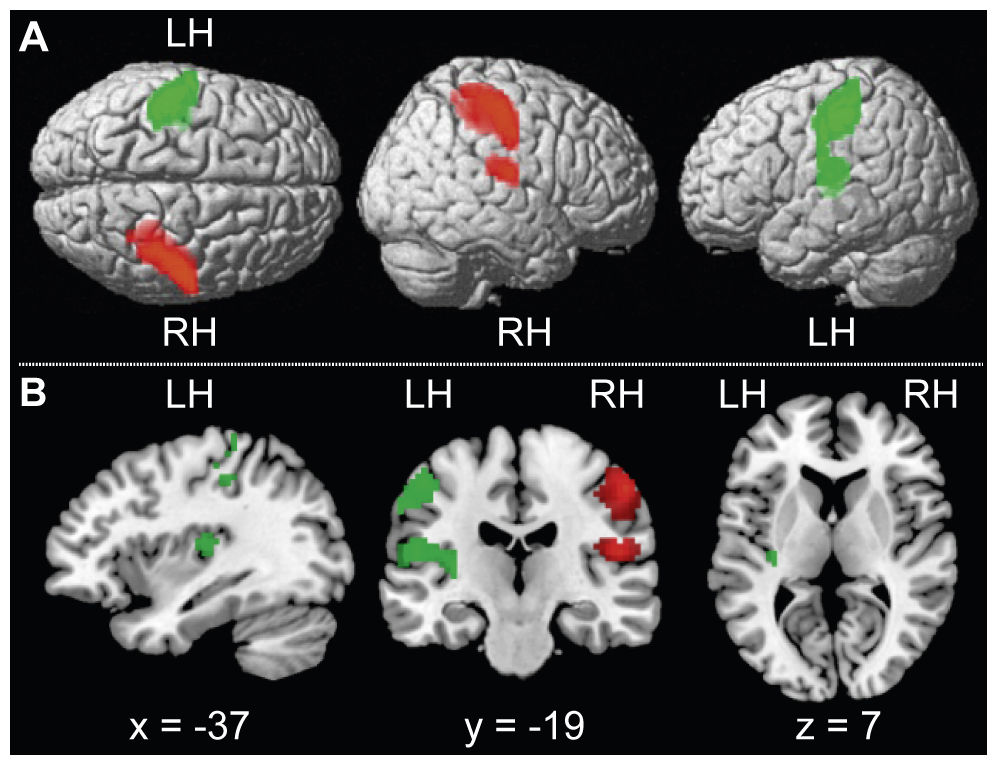
**A** Activation maps for the comparisons target-absent AL > AR and target-absent AR > AL, thresholded at p < .001 uncorrected. Both contrasts revealed increased activity in the contralateral primary and secondary somatosensory cortices. Attention to the right hand resulted in additional activation in left posterior insula. In target-absent AL > AR, right SI is revealed (red) with the MNI coordinates of +54, -19, +49 (x, y, z) for the peak activation as well as right SII with peak activation at +48, -16, +13 (x, y, z). In target-absent AR > AL, left SI is revealed (green) with the MNI coordinates of -45, -28, +49 for the peak activation. Moreover, left SII is significantly activated with peak activation at -48,-22,+16 (x, y, z) as well as left posterior insula with peak activation is at -33, -19, +10 (x, y, z). The contrast maps have been superimposed on an SPM template. From left to right, dorsal and lateral views of right and left hemisphere are depicted. Abbreviation for the left and right hemisphere is LH and RH, respectively. **B** Activation maps for the contrast target-absent AL > AR in green and target-absent AR > AL in red, thresholded at p < .001, uncorrected. Activity in the left primary somatosensory cortex is depicted in the upper cluster of the first two pictures. In the lower cluster the activation over the left posterior insula and left secondary somatosensory cortex is shown. Activity in the posterior insula can also be seen in the third image. From left to right sagittal, coronal and horizontal slices are depicted. Abbreviation for the left and right hemisphere is LH and RH, respectively.

**Figure 3:**
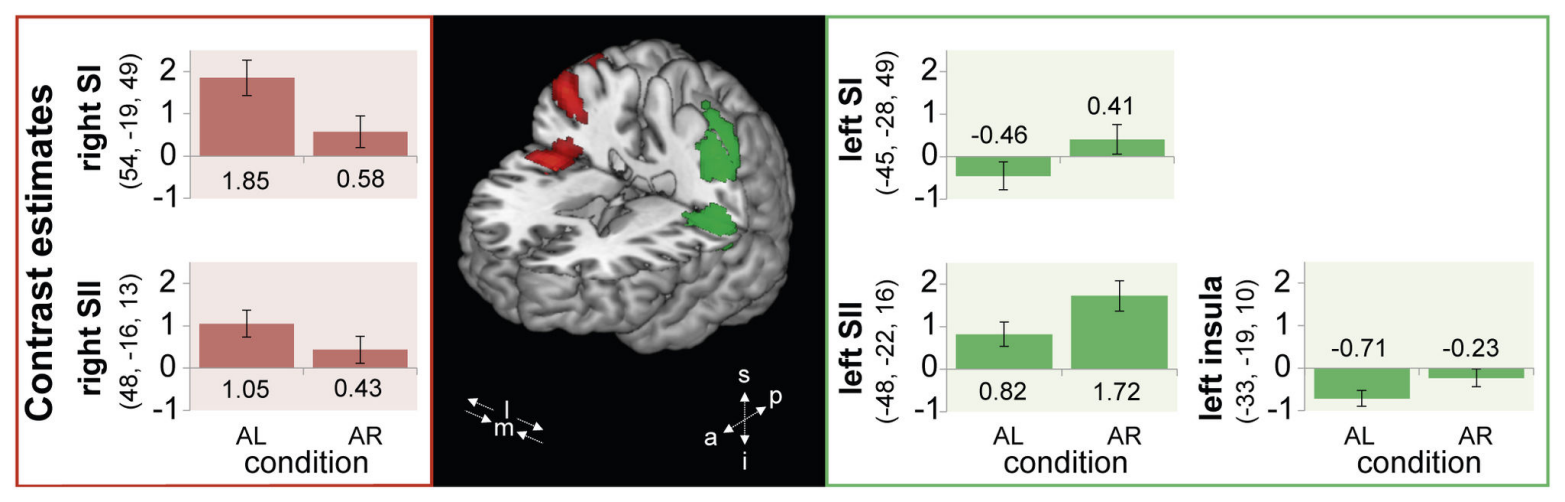
Contrast estimates for peak activations across the two experimental conditions, target-absent AL and AR. Error bars indicate the standard error of the mean, and color coding is equivalent to Figure 2. Red and green clusters are thresholded at p < .001, uncorrected. Abbreviations of the coordinate systems: m = medial, l = lateral; s =superior, i = inferior, a = anterior, p = posterior.

The contrast target-absent AL > AR revealed a significant BOLD activity over right SI covering the upper genu (also referred to as hand knob). In fact, 34.6% of the cluster was covering BA 2, 26.9% was covering BA 1, 23.8% was covering BA 3b, and 5.6% was covering BA 3a. The overall maximal activation is located at x = +54, y = -19, z= +49 in MNI space (*t*(18) = 7.38). Its location is assigned to BA 1 with a probability of 100% for this area at that specific location. Moreover, significant BOLD activity was revealed over right SII, with 52.8% of the cluster covering parietal operculum (OP) 1, 17.5% covering OP 4, 15.9% covering OP 3, and 8.4% covering OP 2 (see also Eickhoff et al. [62] for more details on the human parietal operculum). The maximal activity was observed in OP 1 with a probability of 70% for this area at that specific location (*t*(18) = 8.00, x = +48, y = -16, z = +13). Statistical parametric maps depicting the activated regions are illustrated in Figure 2A and B in red. Bar graphs in Figure 3 show contrast estimates at peak activities for both conditions. As can be seen in the red bar graph, the effect in right SI and SII were both driven by an increased activity in the respective attend condition (AL).

#### Effects of paying attention to the right index finger

The contrast target-absent AR > AL shows a large activation cluster over left somatosensory cortices (see Figure 2A and 2B, green cluster). This large activation comprised: (i) SI covering the hand knob, (ii) SII and (iii) posterior insula. Addressing the activation in SI more closely, we observed that the maximal activation was located in BA 2 with a probability of 70% for this area at that specific location (*t*(18) = 6.99, x = -45, y = -28, z = +49 in MNI space). Moreover, 33.2% of the cluster covered BA 2, 30.5% BA 1, and 22.9% BA 3b. With respect to SII, maximal activation was observed in OP 1 with a probability of 80% for this area at that specific location (*t*(18) = 5.41, x = -48, y = -22, z = +16 in MNI space). Within SII, 63.1% of the activation could be assigned to OP 1, 15.9% to OP 2 and 5.2% to OP 3. With respect to the insula (shown in Figure 2B in the horizontal view on the right), 55.9% of the activation as well as maximal activation was observed in granular layer 2 (*t*(18) = 5.73, x = -33, y = -19, z = +10); 17.1% of the activation was observed in granular layer 1. The activation pattern for left SI, SII, and insular cortex is shown in green in Figure 3. In contrast to the right hemisphere, we observe in the left hemisphere a diverse activation pattern in the different brain regions. Comparable to right SII, the effect in left SII is driven by an increased activity in the respective attend condition (AR). The effect in left SI is related to a slight increase in activity in the attend condition as well as a slight decrease in activity in the respective distraction condition (AL). In contrast, the effect in left posterior insula cortex is exclusively evoked by a relative de-activation in the tactile distraction condition (AL).

### Correlation analyses

**Figure 4:**
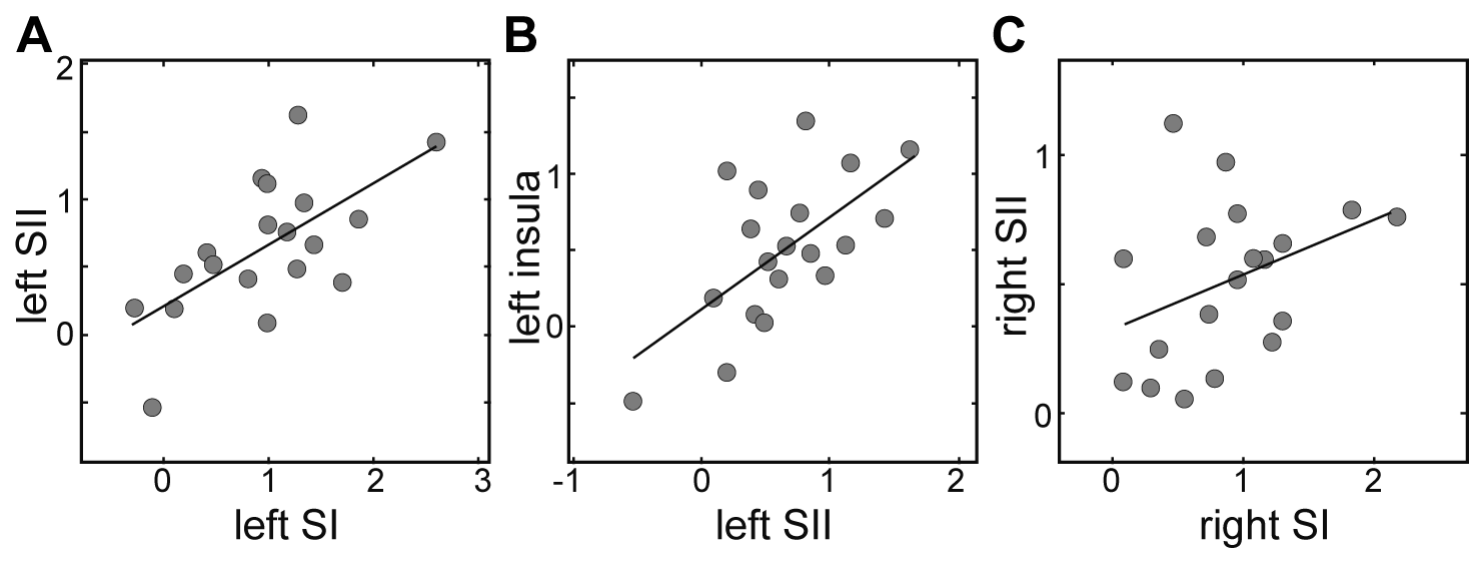
Relationship between the activation values (eigenvariate values) of different brain regions. The correlation between left SI and left SII (**A**), left SII and left insula (**B**), and right SI and right SII (only trend for correlation) (**C**) is depicted. Each circle represents the data of one participant and a trend line is shown to accentuate the correlation.

As can be seen in Figure 4A, there was a highly significant positive linear relationship between the attention effect in left SI and left SII, r = .64, p = .003. Accordingly, left SI and SII share 41.16% of variation in brain activity (R^2^ in percentage). Additionally, there was a highly significant correlation between activity in left SII and left insula (Figure 4B), r = .63, p = .004. Thus, left SII and insula share 40.12% of variation in brain activity. Furthermore, as can be seen in Figure 4C, a trend for a correlation between activity in right SI and right SII was revealed, rho = .41, p = .086. No significant correlation was found between activity in left SI and right SI (rho = .32, p = .180). Similarly, no significant correlation was found between left SII and right SII (rho = -.22, p = .369).

## Discussion

Only a few studies have explored neural mechanisms of sustained spatial attention under conditions of ongoing stimulation (>1 s) to both hands. Contrary to studies that presented transient stimuli with long inter-stimulus intervals (often ≥ 1 s) to only one hand, continuous stimulation to both hands mimics the everyday experience of permanent stimulus input more closely. The aim of the present fMRI study was to provide further insights into brain regions involved in sustained somatosensory spatial attention and their correlational influence on each other. We found that attending to the stimulation of the left or right index finger resulted in increased activity in contralateral SI, mainly covering BA 1, 2, and 3b. Moreover, we found attentional modulation in SII contralateral to the to-be-attended hand as well as modulation in left posterior insula when attention was paid to the right (dominant) hand. Interestingly, most of the effects were driven by an increased activity in the respective attend condition (paying attention to the contralateral hand). Only the effects in left SI and insular cortex show a different pattern. In left SI, we revealed not only a small increase in activation when attention was paid to the contralateral hand but also a small decrease in activation when attention was paid to the ipsilateral hand (i.e., the tactile distraction condition). In contrast, the effect in left posterior insula was exclusively driven by a relative decrease in activation in the tactile distraction condition (AL). Finally, correlation analyses indicate a linear relationship between attention effects in intrahemispheric somatosensory cortices.

In the current study, participants had to attend to the stimulation of one hand and to detect target events that were infrequently included in the stimulation. These target events were important in order to keep participants focused on the task and to obtain a rough measure of subjects’ sensitivity, vigilance state, and compliance. Behavioral data show no difference in target detection or false alarm rate between the right and left hand, thus implying that the task was equally difficult for both hands. This was a prerequisite for comparing the two conditions against each other in the fMRI analysis. The overall detection rate of 61.32% and the small false alarm rate of 0.04% clearly show that the task was difficult but not impossible. 

With respect to attentional modulation in contralateral SI, the current study supplements previous findings on tactile attention in different ways. First, this is one of the first studies describing area-specific attentional modulation within SI. Peak activations were found in BA 1 and BA 2 for the left and right hand condition, respectively. Especially BA 1 is known to respond to cutaneous input and also Hyvärinen and colleagues (1980) showed that attentional modulation occurred predominantly in BA 1 [40]. Besides, BA 2 is also known to have some cutaneous sensibility [52]. Firstly, there is cortical input from areas 3b and 1 to area 2 [63]. Secondly, there is another source of cutaneous information in area 2 which comes from a very sparse but direct thalamic input [63]. Moreover, in contrast to area 1 and 3b, area 2 is also known to be more sensitive to more complex cutaneous stimuli and active tactile discrimination tasks [54-56]. Since in the present study, participants received a relatively complex tactile stimulation requiring focused attention to detect targets, attentional modulation in area 2 might be crucial for a successful performance. Additionally, attention to tactile stimuli that were presented to the finger might promote proprioceptive perception, which is strongly associated with BA2. In addition to BA 1 and 2, attentional modulation in SI was also substantially revealed in BA 3b, also referred to as SI proper [64]. This region is primarily activated by mechanical stimuli, has the largest representation of the fingers, and is known to receive most thalamic projections [64]. Contrary to the present study, Hyvärinen and colleagues (1980) showed that attentional modulation occurred relatively sparsely in BA 3b [40]. However, they recorded neurons from extracellular microelectrodes in three monkeys and revealed that only 16% of recorded neurons in SI were affected by attentive behavior. Comparable to our study, they also investigated sustained attention to vibrotactile stimuli, but the different outcome with respect to BA 3b presumably results from the different experimental recordings and the chosen control condition. In contrast to the present active tactile distraction condition (i.e. attending to the ipsilateral hand), a passive ignore condition was chosen in the study of Hyvärinen and colleagues (1980).

Our findings strongly support those EEG and imaging studies that show attentional modulation in SI [3,35,36]. Moreover, our methodological approach further substantiates the hypothesis of Johansen-Berg and colleagues (2000) [47]. They assumed that two factors might be substantial in detecting attentional effects in SI: Firstly, an *active* distractor task in the respective ignore/control condition seems to better control a participant’s attention and directs attention away from the tactile input. Secondly, a region of interest approach seems to be helpful by taking a greater variability of SI responses into account and restricting the analyses to the somatosensory area. Interestingly, few researchers have thought of using an active distractor task within the tactile domain [3,48,49], although we experience *permanent or ongoing* tactile input to *both* hands in everyday life. Besides, many of these studies showing SI effects either investigated transient attention to brief tactile events, or investigated “sustained” attention by applying blocks of transient tactile events to one hand only. In these cases, attention conditions were either compared to ignore conditions, or attentional resources were withdrawn from the tactile domain by cognitive tasks (e.g., mental arithmetic or visual distraction). In the studies of Eimer and colleagues (2003), attention was not withdrawn to other modalities, but remained in the tactile domain [3,39]. For example, in one of their EEG studies, attention was spatially manipulated within one hand [39]. In another study, they presented tactile stimulation bimanually and manipulated transient as well as sustained spatial attention between the two hands [3]. Their results indicate that sustained attention modulates tactile processing as early as in SI, while effects of transient attention become apparent beyond SI. Moreover, Meador and colleagues (2002) also used a bimanual tactile stimulation paradigm and compared attend right and attend left hand conditions [49]. They revealed that attention to the right hand increased activation in the left primary somatosensory cortex, and that attention to the left hand increased activation in a distributed network including somatosensory, frontal, and occipital regions over both cerebral hemispheres. Especially, this widespread pattern for the left hand is in contrast to the current findings, and it can presumably be explained by the two different tasks: recognizing digits written on a subject’s palm (Meador et al., 2002) vs. sustained attention and detection of targets in a vibrotactile stream. In considering these studies we can conclude that this is one of the first fMRI studies that uses spatial manipulation of attention within the tactile modality rather than withdrawing attentional resources from that modality by cognitive tasks (e.g., mental arithmetic). To sum up, our imaging study further substantiates previous studies by showing SI modulation as a function of *sustained spatial attention* to ongoing flutter vibration.

A further supplement can be made to our previous EEG study [32]. The use of a similar design (trial-by-trial cueing) as well as a similar stimulation frequency range allowed a direct comparison between previous and current results. Since we applied the same frequency to both index fingers in the current study, we can definitively exclude the possibility that subjects were using an alternative strategy to perform the task, that is, attending to the feature *vibration frequency* rather than to the *cued location*. Furthermore, it should be emphasized that EEG source analyses of frequency-tagged vibrotactile stimulation has not been validated so far. It has only been shown in the visual modality that EEG source analyses of frequency-tagged flickering stimuli as well as attentional BOLD modulation nicely correspond [65]. Both methods localized areas in the extrastriate visual cortex. With our current study, we are thus able to validate our previous EEG source analyses [32] and confirm that SI mediates sustained spatial attention to vibration.

In addition to the attention effect in SI, we found attentional modulation in SII (mainly OP 1), contralateral to the to-be-attended hand. Moreover, attention to the right (dominant) hand resulted in additional activity in left posterior insula. Interestingly, these activations were not revealed in our previous EEG study [32]. Different reasons might account for this: First, the well-known problem of spatial resolution and volume conduction in EEG that limits the validity of estimated cortical sources in a number of circumstances. Furthermore, although EEG is more sensitive to radial sources, it is almost blind to tangential sources that might be more prominent in SII lying in the upper bank of the sylvian fissure. Finally, in the previous EEG study we needed to stimulate the hands with two different frequencies in order to be able to distinguish the neural responses. This might have resulted in the focused SI pattern. Generally, SII and insula are both known to play a crucial role in somatosensory processing [66-69]. Importantly, attentional modulation in the posterior insula as well as in SII occurred predominantly bilaterally in previous studies [36,38,44,70]. Assuming a comparable attentional modulation of these structures when attending to the right or left hand, one would rather expect a cancellation due to the direct comparison of both sides. However, our results show robust effects in these areas. Interestingly, all of the aforementioned effects were driven by an increased activity in the respective attend condition (i.e., when attending to the contralateral index finger), thus supporting the studies claiming that attention increases neural activity. Only for the effects in left SI and left posterior insula did we reveal a diverse activation pattern. The effect in left SI was not only related to a minor increase in activation when attention was paid to the contralateral hand, but also related to a minor decrease in activation in the respective distraction condition (i.e., when attention was paid to the ipsilateral hand). In contrast, the effect in left posterior insula was solely driven by a relative de-activation in the respective distraction condition (cf. Figure 3). In general, these patterns are comparable to the findings of Sterr et al. (2007) and Burton and colleagues (2008) showing that attention effects can additionally be driven by a relative de-activation in the respective control condition [35,38]. However, in contrast to their studies, we found decreased activation in left SI and posterior insula and not in SII. We think that this divergence in results is the consequence of different experimental control conditions (mental arithmetic in the study of Sterr et al. (2007)). Moreover, the fact that both hands were simultaneously stimulated instead of only one could also have had a substantial impact on the results. Nonetheless, similar to Sterr and colleagues (2007), we assume that this de-activation might reflect an active inhibition of these regions when tactile information is irrelevant.

Considering the differential activation pattern for the right (dominant) hand in left SI and posterior insula, we can only speculate. Given that all our subjects were right-handed, we cannot infer from the present study whether it is unique to the right or the dominant hand. Yet, from previous studies it is known that the left and right hand have different perceptual thresholds for tactile stimuli [71,72]. In the present study we used the same force for left and right hand stimulation, but a possible difference in perceptual thresholds between the left and right hand would have presumably resulted in significant differences in target detection rates, something that we did not observe. Future research is needed to explain the different activation patterns for the right and left hand and the influence of hand dominance.

A further advance of our study is to directly relate the aforementioned attention effects to each other by using correlation analyses. We found a significant correlation between BOLD activity in left SI and left SII. Since there are reciprocal connections between SI and SII [50], it is not clear whether activity in SI influences SII, or whether SII affects SI via feedback projections. In addition, a trend for a correlation between attentional BOLD activity in right SI and right SII could be revealed. Possibly, this did not reach significance because the left hand is not the dominant hand in our participant sample. A highly significant correlation was further revealed in the connected brain regions, left SII and left insular cortex. However, this finding should be interpreted with caution as spatial smoothing was applied and the peaks in the respective regions are located within one large cluster spanning across these closely connected brain areas. Although there are direct transcallosal connections [50], we found no significant correlation between left and right SII. Moreover, a recent paper by Ragert et al. (2011) revealed an interhemispheric information transfer between left and right SI [73], suggesting a further possible transcallosal interaction. Yet, this could not be supported by our correlation analysis. Taking these findings into consideration, correlation analyses suggest that there is a direct linear relationship between the attention effects in SI and SII within one hemisphere but not across hemispheres.

It should be noted that although we found attentional modulation as early as in SI, it does not imply that our findings refute those EEG studies revealing attentional modulation only in a later time window corresponding to SII. Different experimental recordings might cause different results. Moreover, as mentioned above, the current fMRI study does not disentangle whether the attention effects in SI influence SII or vice versa. Especially, if the first (significant) attentional modulation becomes apparent in SII, and the feedback projections from SII to SI were to be the main cause for the attentional modulation in SI, attention effects in an early time window that clearly corresponds to SI would not be detectable in EEG measurements.

Generally, we think that our findings can also be generalized to a wider frequency range. However, we assume there to be some limitations. In our opinion, two crucial aspects have to be fulfilled: *Sustained attention* and *continuous* tactile stimulation. We assume that attention can fluctuate and might not remain continuous on tactile events as soon as the applied frequency range is too low (<1-2 Hz). However with higher frequencies, even outside the flutter range, we can imagine comparable results. Nonetheless, we focused on a frequency in the flutter range, and thus future research is needed to explore the impact of the applied frequency.

In summary, the current study provides valuable insights into brain areas involved in sustained somatosensory spatial attention. In particular, we showed that activity in contralateral SI and SII is subject to attentional modulation. With respect to SI, we described area-specific attentional modulation and revealed that attentional modulation is especially pronounced in BA 1, 2 and 3b. Interestingly, we showed that attention to the right (dominant) hand recruited an additional area, the contralateral posterior insula. Essentially, the effects were driven by an increased activity when attention was paid to the contralateral hand. Yet, in left insula and left SI an (additional) de-activation in the tactile distraction condition was revealed, possibly reflecting an active inhibition when tactile stimuli are task-irrelevant. Finally, we found a correlation between the attention effects in intrahemispheric somatosensory cortices, which helps to elucidate the interplay between SI and SII when attention is paid to specific body locations. All in all, our results promote future research in the realm of sustained attention to continuous vibrotactile stimulation in the range of flutter.
