# Correction: Sustained Spatial Attention to Vibrotactile Stimulation in the Flutter Range: Relevant Brain Regions and Their Interaction

**DOI:** 10.1371/journal.pone.0099556

**Published:** 2014-05-30

**Authors:** The PLOS ONE Staff

The third author's name is spelled incorrectly. The correct name is: Sabrina D. Thiel. The correct citation is: Goltz D, Pleger B, Thiel SD, Villringer A, Müller MM (2013) Sustained Spatial Attention to Vibrotactile Stimulation in the Flutter Range: Relevant Brain Regions and Their Interaction. PLoS ONE 8(12): e84196. doi:10.1371/journal.pone.0084196
